# Correction: Biofuel production from waste residuals: comprehensive insights into biomass conversion technologies and engineered biochar applications

**DOI:** 10.1039/d5ra90054a

**Published:** 2025-05-16

**Authors:** Esraa M. El-Fawal, Ahmed M. A. El Naggar, Adel A. El-Zahhar, Majed M. Alghamdi, Asmaa S. Morshedy, Hussien A. El Sayed, Ard elshifa M. E. Mohammed

**Affiliations:** a Egyptian Petroleum Research Institute (EPRI) 1 Ahmed El-Zomor St. Nasr City Cairo Egypt drmeto1979@yahoo.com; b Department of Chemistry, Faculty of Science, King Khalid University Abha 9004 Saudi Arabia; c Department of Chemistry, College of Science, Qassim University Al-Qassim Saudi Arabia

## Abstract

Correction for ‘Biofuel production from waste residuals: comprehensive insights into biomass conversion technologies and engineered biochar applications’ by Esraa M. El-Fawal *et al.*, *RSC Adv.*, 2025, **15**, 11942–11974, https://doi.org/10.1039/D5RA00857C.

The authors regret that the name of one of the authors (Majed M. Alghamdi) was shown incorrectly in the original author list and author biography section. The corrected spelling is shown herein.

The Royal Society of Chemistry apologises for these errors and any consequent inconvenience to authors and readers.

